# Maternal early warning scores shown to be methodologically weak and at high risk of bias

**DOI:** 10.1016/j.jclinepi.2025.111833

**Published:** 2025-05-19

**Authors:** Mae Chester-Jones, Shona Kirtley, Fema Er, Vidoushee Jogarah, Yu Qiao, Ruth Tunn, Naomi Vides, Peter J. Watkinson, Marian Knight, Stephen Gerry, Gary S. Collins

**Affiliations:** a Centre for Statistics in Medicine, Nuffield Department of Orthopaedics, Rheumatology and Musculoskeletal Sciences, Botnar Research Centre, University of Oxford, Oxford, UK; b National Perinatal Epidemiology Unit, Nuffield Department of Population Health, University of Oxford, Oxford, UK; c Kadoorie Centre for Critical Care Research and Education, Nuffield Department of Clinical Neurosciences, University of Oxford, Oxford University Hospital NHS Trust, NIHR Biomedical Research Centre Oxford, Oxford, UK

**Keywords:** Prognosis study, Maternal early warning score, Clinical prediction model, Obstetrics, Maternal morbidity, Maternal mortality, Systematic review, Pregnancy, Missing data

## Abstract

**Objectives:**

To systematically review and critically appraise the methodology of developing modified obstetric early warning scores (MOEWSs).

**Study Design and Setting:**

We searched Medline, CINAHL, EMBASE, and the Web of Science for MOEWS studies published between January 1, 2000, and December 31, 2022. Eligible studies included models predicting maternal death, intensive care unit (ICU) admission, and/or a composite of two or more maternal morbidities occurring in a hospital setting in women of any gestational age and up to 1 week after the end of pregnancy. Models were critically appraised using the Prediction Model Risk of Bias Assessment Tool (PROBAST) and adherence to the transparent reporting of prediction models (TRIPOD).

**Results:**

20 studies were included: five (25%) were model development studies, five (25%) were model development and validation, and ten (50%) were validation only. Four development studies used statistical methods, and the remaining six studies used clinical consensus (ie, expert opinion). The four data-driven model development studies did not address key statistical challenges, such as repeated measures or missing data, nor did they assess the performance adequately or dataset characteristics clearly. All but one study (95%) were rated at high risk of bias due to data sources, poor reporting, and analysis limitations. The fifteen validation studies were poorly reported and eleven (73%) were at high risk of bias. None of the data-driven models were independently validated, a key step toward implementation.

**Conclusion:**

There is a lack of MOEWSs developed using methods that follow recommended statistical guidelines. Substantial problems with the methodological quality of included development and validation studies, along with high risk of bias,indicating published scores could perform poorly or be potentially harmful if used in clinical practice. Future work should address handling missing data and repeated measures and consider how an MOEWS will perform in different populations and key subgroups.

## 1 Introduction

Maternal deaths have increased in Europe and North America [1], with the United States having the highest among developed countries [2], followed by New Zealand, France, and the UK. In both the UK and United States, Black women are three times more likely to die during or up to 6 weeks after pregnancy than White women [2,3]. Although maternal death is rare in places like the UK, for every woman who dies, 100 experience a life-threatening complication during pregnancy [4]. These are severe maternal morbidities, such as sepsis or hemorrhage, that have a long-term health impact [5]. Research suggests that up to two-thirds of maternal deaths may be preventable if deterioration is identified early [6].

Modified obstetric early warning scores (MOEWSs) are clinical prediction models (CPMs) used to identify physical deterioration in pregnant or postpartum women, before they have a life-threatening event. Vital signs such as systolic blood pressure (SBP), respiratory rate, oxygen saturation, heart rate, and temperature are collected at bedside, assigned a score based on their abnormality and summed to give an overall score. Unlike CPMs in other clinical areas that are rarely implemented [7], MOEWSs are in routine use. However, a lack of standardization in how they are operationalised has led to multiple, inconsistent and variable MOEWSs [8], many developed through clinical consensus rather than statistical modeling [8,9]. National scores implemented in Ireland (2014 [10]), Scotland (2018 [11]), and New Zealand (2018 [12]) were developed through clinical consensus, while a new national MOEWS developed using statistical modeling is being implemented across England [13].

The stages of CPM production includes development and internal validation, external validation, and implementation [14,15]. Numerous guidelines exist for CPM development and validation [16,17] that recommend reporting sample size calculations [18–21], predictive performance [14,15,21,22], and addressing missing data at all stages. It is recommended to use multiple imputation during development and explore the missingness mechanism [16,17]. However, the best methods to handle missingness at implementation are unclear [23–25] and any methods to handle missing data at implementation should be assessed at validation [24]. Reviews of CPMs have found that missing data are frequently poorly handled and reported [13,26–28]. Developing an MOEWSs is uniquely challenging due to the dynamic nature of their use throughout pregnancy and the postpartum period. This creates records with multiple measurements (or observation sets) of vital signs, and it is unclear what the best method is to handle these repeated measures [29,30].

A previous review of MOEWSs reported good predictive accuracy [9]; however, the authors did not assess the methodological quality of the studies. Another review found high variation in the trigger thresholds across 147 MOEWSs in use in UK hospitals [8]. A review of early warning scores (EWSs) in the general hospital population have also concluded that many are insufficiently reported and that the development is of poor methodological quality [31]. A review of obstetric CPMs found studies to be incompletely reported and the model quality too poor to be deployed [32].

As maternal death is a rare event and difficult to predict, it can be easier to predict severe maternal morbidities or intensive care unit (ICU) admission (a proxy for maternal deterioration). To identify studies for this review, we defined an MOEWS as a score comprising at least two vital sign predictors used to identify women at risk of deteriorating and experiencing maternal death, ICU admission, or a composite of two or more severe maternal morbidity outcomes (sepsis or systematic infection, severe postpartum haemorrhage, severe preeclampsia or eclampsia, or ruptured uterus). We included these morbidities as they are defined as severe maternal complications by the World Health Organization (WHO) [5].

The primary aim of this systematic review was to critically appraise the methodological conduct, examine the completeness of reporting, and assess risk of bias of studies describing the development or external validation of MOEWS during pregnancy and the immediate postpartum period. A secondary aim was to identify national documented MOEWS that are used in the UK and Ireland.

## 2 Methods

We registered this systematic review (16 February 2023) on the International Prospective Register of Systematic Reviews (PROSPERO CRD42023396218). The systematic review was carried out and reported in accordance with two published guidelines: the Critical Appraisal and Data Extraction for Systematic Reviews of Prediction Modeling Studies (CHARMS) checklist [33] and the Transparent Reporting of multivariable prediction models for individual prognosis or diagnosis: checklist for systematic reviews and meta-analyses (TRIPOD-SRMA [34]).

### 2.1 Selection criteria

Studies were assessed for the inclusion and exclusion criteria listed below. We also included the national Scottish and Irish scores for comparison due to their importance in the field as standardised and implemented MOEWSs although they are not from published studies. The new UK maternity early warning score was unpublished at the time of the search and was not included [13].

### 2.2 Inclusion criteria

- The study described development or external validation of one or more MOEWSs, defined as a score used to identify women at risk of deteriorating and experiencing maternal death, ICU admission, or a composite of two or more maternal morbidity outcomes
- The MOEWSs includes at least two vital sign predictor variables to produce an individualised estimate of risk or indicator of deterioration (eg, a score)
- The MOEWSs was developed to be used during pregnancy or up to 1 week after the end of pregnancy
- The MOEWSs was developed to be used repeatedly (ie, applied at multiple timepoints throughout pregnancy and immediately after pregnancy) rather than at a single timepoint

### 2.3 Exclusion criteria

- The MOEWS was used to solely predict risk of neonatal outcomes
- The MOEWS used mainly fetal predictors
- The MOEWS was intended for use only outside of a hospital setting
- The MOEWS was developed for use in particular subsets of women, for example women with specific diseases, critically ill women, or women from low resource settings
- Papers not written in English
- Papers published before 2000
- Preprints, journal or conference abstracts, and trial protocols

#### 2.3.1 Study search and selection

The Medline (via OVID), CINAHL (via EBSCoHost), EMBASE (via OVID), and Web of Science (via Clarivate) databases were searched on 21 March 2023 for studies published between January 1, 2000, and December 31, 2022.

The search strategy comprised relevant controlled vocabulary headings (eg, Medical Subject Headings or EMBASE Subject headings) and free-text variants for early warning score terms, development or validation terms, prognostic or prediction modeling terms, and pregnancy-related terms. Aside from the publication date limits described above no other limits were applied to the search in CINAHL, Medline, or Web of Science. An additional limit to exclude conference abstracts was applied to the EMBASE search. The full search strategy for each database, developed by a senior information specialist (S.K.), can be found in Appendix B. Deduplication was performed in EndNote [35], and abstracts were screened using Rayyan [36] by M.C.J.

#### 2.3.2 Data extraction

Full-text screening and data extraction were carried out in duplicate by six independent reviewers (M.C.J., F.E., V.J., Y.Q., R.T., and N.V.), with discrepancies resolved by S.G. or G.S.C. Data from the included studies were captured using a data extraction form administered through REDCap (Research Electronic Data Capture) hosted by the University of Oxford [37,38]. The form was piloted on five papers. Items extracted included.

- Type of study (eg, development, external validation, both)
- Characteristics of the population
- Time period of the models intended use (antenatal period, postpartum period)
- Sample size (and number of outcome events)
- Model building steps and approaches
- The handling of continuous predictors
- Whether internal validation was undertaken and the approaches used
- Model performance measures
- The handling of missing data
- Details of any external validation undertaken
- Open science practices

#### 2.3.3 Article publication information and risk of bias

Each study was assessed for adherence to the TRIPOD reporting guideline [16], and risk of bias was rated as “high,” “low”, or “unclear’ using the Prediction Model Risk of Bias Assessment Tool (PROBAST) tool [39].

#### 2.3.4 Evidence synthesis

Extracted data were summarized using descriptive statistics, visual plots, and with a narrative synthesis.

## 3 Results

### 3.1 Summary of included studies

**Figure 1:**
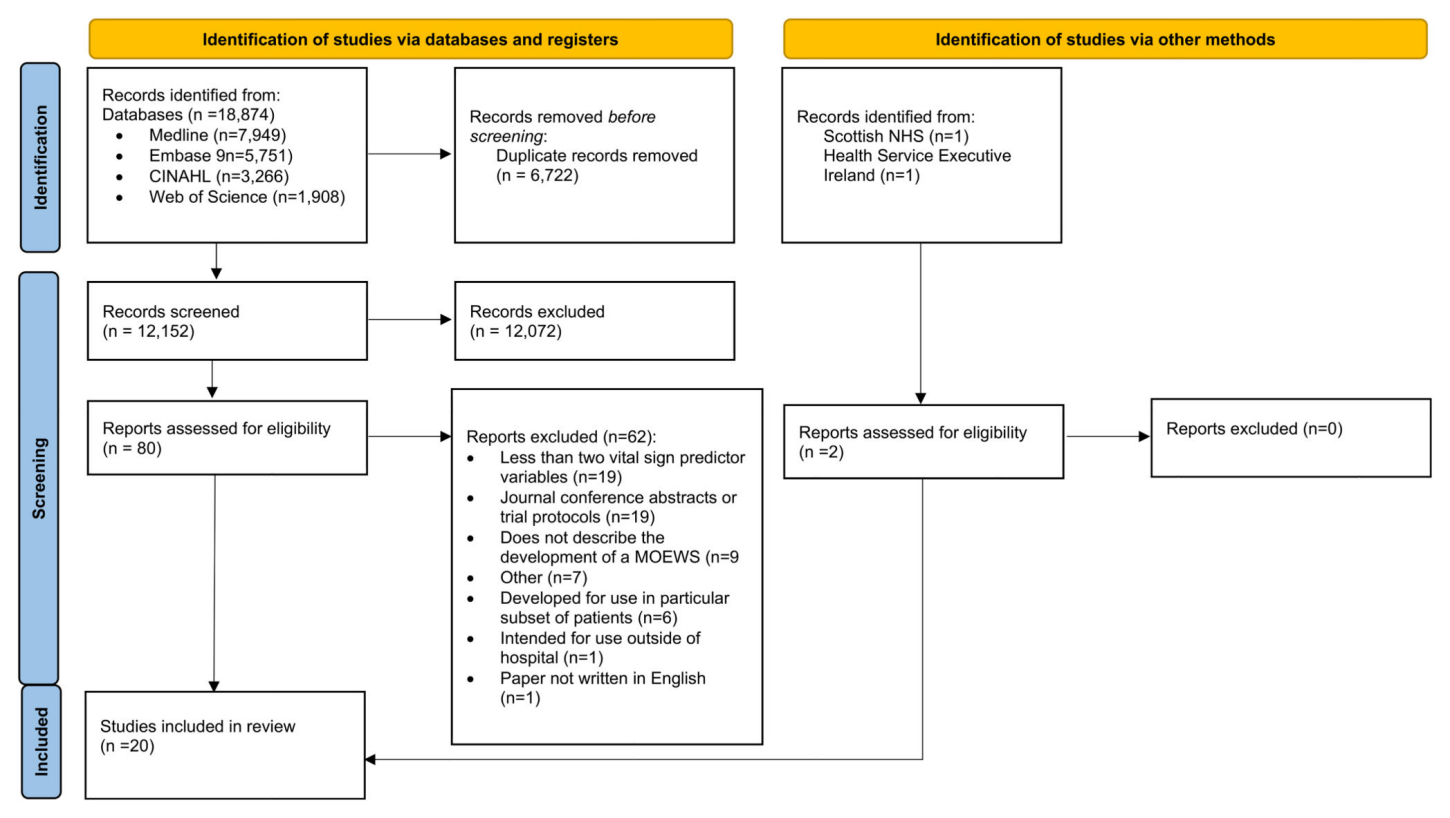
Flow chart of studies included in the review.

The search identified 18,874 studies (Fig 1). After removing 6722 duplicates, 12,152 abstracts were screened, with 80 undergoing full-text screening where 62 studies were excluded. A total of 18 published studies met the inclusion criteria, which we combined with the Irish and Scottish national MOEWSs to leave a total of 20 articles for full-text review and data extraction.

All 20 included studies were published between 2009 and 2022; 10 were model development studies [10,11,40−47], including five studies that included external validation [40,42−44,47] and 10 external validation-only studies [48−57]. Studies were from the United States (*n* = 3), Scotland, Ireland, Finland, Bangladesh, Canada, India, and Spain (all *n* = 1). The external validation-only studies were from the United States (*n* = 7), India (*n* = 2), and England (*n* = 1).

Fewer than half of the studies were published open access, Appendix Table A-1 [7]. Only two studies mentioned following a reporting guideline [43,44].Three studies provided a data-sharing statement [44,46,49]; two of these studies provided details on how to access the data [44,46]. None of the included studies referenced a study protocol or a study registration.

### 3.2 Studies describing the development of an MOEWS

#### 3.2.1 Study design and participants

The development studies methodology was categorised as using clinical consensus (ie, a team of experts deciding the thresholds) or a data-driven approach (ie, using statistical methods). Six development studies (60%) used clinical consensus to determine which vital signs to include and their respective thresholds: the Irish and Scottish national scores (Table 1) [10,11], the maternal early warning criteria (MEWC) [41], the maternal early warning trigger (MEWT) [42], and two modified scores [44,47]. The remaining four studies used a data-driven approach [40,43,45,46].

The four data-driven model development studies included two logistic regression models [40,43], a deep learning model [45] and a risk decision tree [46], developed using case-control, retrospective, simulated and unreported datasets, respectively, Table 2. Three studies reported sample size of *n* = 184 [43], *n* = 1218 [46], and *n* = 176,731 [40], with no formal sample size calculations. Two studies reported maternal age and gestational age at hospital admission [40,43], but no study reported a breakdown of ethnicity. Final model coefficients were partially reported in two studies, Table A-3.

Seven studies developed a new MOEWS [10,11,40–42,45,46], and three studies (30%) modified existing scores [43,44,47]. One study [44] modified the MEWC [41], and two studies [43,47] modified an example MOEWS from the Confidential Enquiry into Maternal and Child Health (CEMACH report 2003-2005) [58]. This chart was not developed by the authors, but our review found it has been modified or validated multiple times and will be referred to as the CEMACH MOEWS throughout.

#### 3.2.2 Development: outcomes

The outcomes predicted included composite of maternal morbidities (*n* = 4, [40,42,44,47]), general maternal deterioration (*n* = 3, [10,11,41]), categories of risk of complication (*n* = 2, [45,46]), and ICU admission (*n* = 1, [43]), shown in Table 2 with the full list of outcomes in Table A-4. The time window of prediction was during hospital admission, as defined in the protocol. Two data driven studies reported the number of events, see Table A-4 [40,43].

#### 3.2.3 Development: predictors

The MOEWSs included between 4 and 35 predictors with a median of nine predictors, see Table A-5. The most common predictors were SBP (*n* = 10), diastolic blood pressure (DBP) (*n* = 8), heart rate (*n* = 8), temperature (*n* = 7), respiratory rate (*n* = 7), oxygen saturation (*n* = 6), urine output (*n* = 4), and maternal age (*n* = 3), see Table 2. All predictors are described in Table A-2.

#### 3.2.4 Development: missing data

Two studies addressed missing data during development [40,43], see Table A-6. Both studies described the proportion of missingness and approach to handling missing data, complete case analysis (CCA) [43] and last observation carried forward (LOCF), imputation to normal, missing indicators, and trajectories [40]. Only one reported the missing data mechanism (missing-at-random) [43]. No studies discussed handling missing data at the point of implementation.

#### 3.2.5 Development: multiple observations

Of the four data-driven models, no studies reported the number of observation sets per woman, see Table A-4. It was unclear for two studies how multiple observation sets per woman were handled in the analysis [45,46].

#### 3.2.6 Development: model performance and internal validation

Of the four data-driven models, one study [43] reported the apparent model performance, see Table A-7. Three studies carried out internal validation, where split sample [40,46] or cross-validation was used [45], see Table A-8. None of the studies reported the results of the internal validation.

#### 3.2.7 Validation: study design and participants

Fifteen validation studies were identified, of which five were part of the model development and ten external validation-only studies (Table 3). Ten studies used existing data, and five prospectively collected new data. Stage of pregnancy included any stage (*n* = 4), the antepartum and postpartum (excluding intrapartum or labor) (*n* = 3), intrapartum and postpartum (*n* = 3), intrapartum (*n* = 2), the early postpartum (*n* = 2), or antepartum (*n* = 1), Table A-9. No study reported the number of events per stage.

The sample sizes used to validate the scores ranged between 123 and 54,429 pregnant women, see Table 3. No study reported all of maternal age, ethnicity, and gestational age: maternal age was reported in 12 studies (80%) and gestational age and ethnicity was reported in six studies. Ten studies described using multiple observations, but the number of observation sets per woman was not reported.

The most common score validated, or modified and validated, was the CEMACH MOEWS [60] by eight studies [43,47,49,50,52,53,55,57], although this did not meet our definition of an MOEWS development study (see section 3.2.1). The MEWT [42] and MEWC [41], which were developed using clinical consensus, were externally validated multiple times; the MEWT six times [42,49−51,54,55]) and the MEWC five times [44,48−50,55]). One study validated a model developed using statistical modeling (the authors’ own model [40]), and 14 studies (93%) validated scores developed by clinical consensus, Table 3.

#### 3.2.8 Validation: outcomes

The outcomes were mainly composite (*n* = 13), with two studies predicting ICU admission (*n* = 2, [43,54]), shown in Table 3 with the full list of observations in Table A-9. The number of events was reported completely in thirteen studies and partially in two studies, where they did not provide a breakdown for each component of the composite outcome, Table A-10 [40].

#### 3.2.9 Validation: assessment of predictive performance

Fourteen studies (93%) validated simple MOEWS charts and most commonly reported the sensitivity (14 studies), specificity (all 15 studies), negative predictive value (11 studies), and positive predictive value (12 studies), see Table A-12. Four studies (26%) assessed discrimination with the c-statistic [40,49,56,57]. Calibration was assessed in one study [36].

#### 3.2.10 Validation: missing data

Three validation studies (20%) mentioned missing data [40,43,52], see Table A-11. The proportion of missingness in the predictors was reported in two studies [43,52]. Only one study reported an assumption of the missingness mechanism [43]. The methods to handle missing data were CCA [43], a combination of LOCF, imputation and missing indicators [40], and reconstructing observations from clinical notes [52].

#### 3.2.11 Risk of bias

**Figure 2:**
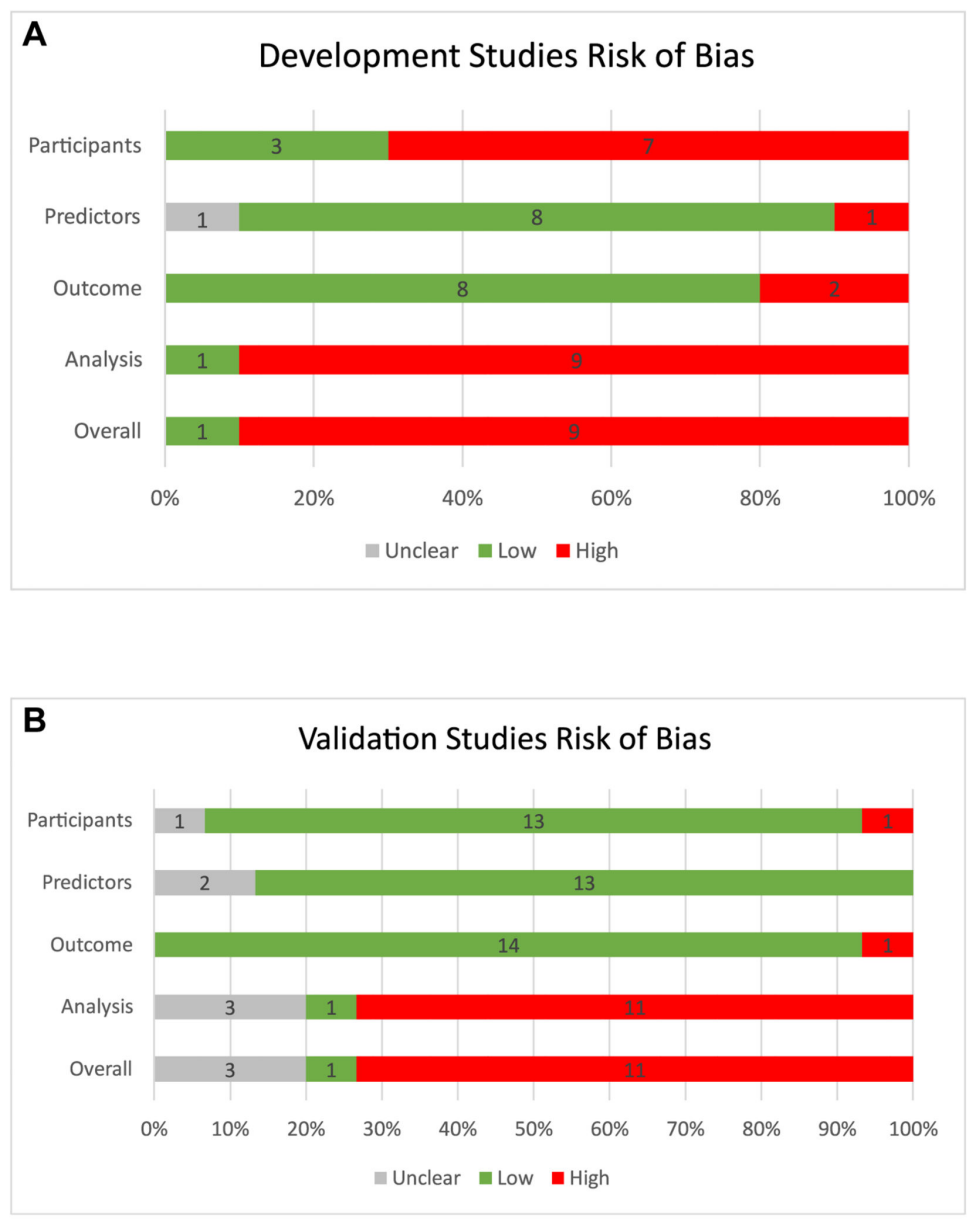
Risk of bias of development (A) and validation (B) studies. The five studies that developed and validated a model were assessed for bias individually for the development and validation methods.

Overall, nine development studies were rated at high risk of bias (90%), Figure 2A. This was either high risk of bias in the participants domain (70%) due to lack of data or high risk of bias in the analysis domain (90%) due to unclear reporting and poor handling of missing data, limited discussion of complexities in the data (ie, handling of repeated measures), and absence of model evaluation.

Most of the validation studies were at low risk of bias for the participants (13/15 87%), predictors (13/15 87%), and outcome domains (14/15 93), Figure 2B. Eleven studies (73%) were rated at high risk of bias in the analysis domain due to a small number of events or not reporting the number of events, inadequate handling of missing data, not providing information on handling data complexities, or not reporting relevant performance measures. Eleven studies (73%) were rated at high risk of bias overall, Figure 2B. One study, Escobar et al [40] was at low risk at bias for both development and validation.

## 4 Discussion

### 4.1 Summary of findings

We examined the design, methodological conduct, and reporting of 20 studies, describing the development or validation of an MOEWS. Most studies developed simple scores with either single or aggregate trigger thresholds, assuming each variable has the same predictive value. Only four studies developed an MOEWS using a data-driven approach, and almost all (3/4) were at high risk of bias. Poor reporting of model performance prevented comparisons of these models. Methods for handling repeated measures and missing data were rarely addressed.

The majority of external validation studies (14/15 93%) validated scores developed using clinical consensus: the MEWC [41], MEWT [42], and CEMACH MOEWS [58]. Due to a lack of scores developed using statistical modeling, and thus no individual risk estimation, important prediction model metrics, such as calibration and discrimination, were rarely reported. Additionally, 66% of validation studies were carried out using historical data collected for a different research purpose.

Missing data were rarely addressed. In the few cases where it was described, most authors relied on the inefficient CCA or impractical methods such as reconstructing missing observations from notes. There was no consideration by any of the studies on missing data at the point of use and the impact this would have on model performance.

The reporting of demographic characteristics was often poor, and investigation of these subgroups was rarely undertaken despite widespread reports that maternal morbidities and death are more likely to affect non-white women. Without the assessment of MOEWSs in these key areas, it is unknown how MOEWSs perform in women most at risk of morbidity and mortality.

Open science practices were rarely followed. One of the benefits of open science is efficiency because it reduces duplication of research. However, the repeated creation of new MOEWS based on clinical consensus rather than data, coupled with the low quality of development studies and the validation of similar MOEWS on comparable datasets, indicates a significant waste of resources. Additionally, the overall quality of the studies was very poor. Comprehensive reporting of methods would enhance reproducibility, allowing for meaningful replication and validation of the models.

### 4.2 Current literature

Previous reviews have assessed the reported predictive accuracy of MOEWS [9], other prognostic models in obstetrics [32], or identified and compared thresholds of MOEWS in use [8], and none have critically appraised the design and methodology of existing published MOEWSs.

### 4.3 Strengths and limitations

The strengths of this research are that it critically assesses and synthesizes important aspects of development and validation of existing MOEWS based on the CHARMS checklist [33] and risk of bias using the PROBAST tool [39].

We note that previous reviews have already found these models to largely be of poor quality [32]. Although 147 charts are in use, many of these scores have never been published in peer-reviewed articles. Consequently, the details behind how these scores were developed and evaluated remain unclear, and no published data exist on them.

We used the WHO definition of severe maternal complications [5] but did not include all clinical conditions associated with life-threatening maternal events. However, we recorded all outcomes identified by our identified models and believe the majority, if not all, of MOEWSs predicting these complications will have been identified with our search.

### 4.4 Recommendations for future research practice

Our review highlighted a gap between model development and model implementation. We recommend adhering to reporting guidelines, such as TRIPOD [16] and TRIPOD + AI [17], and propose the following for future MOEWS development.

#### 4.4.1 Data description

Key demographics associated with severe outcomes were rarely described. As outcomes vary by stage of pregnancy, we recommend:

- Use representative datasets and describe participants characteristics, in particular demographics associated with worse outcomes in the population, and whether the included participants are representative of the target population, including underrepresented groups [3,16,17].
- Report the population characteristics and number of events overall and by antepartum, labor, and postpartum, and assess model performance (discrimination, calibration) within the stages of pregnancy.

#### 4.4.2 Develop MOEWSs using statistical modeling

Vital signs are taken frequently at ad-hoc timepoints and often at multiple hospital admissions, particularly for women with worse outcomes. However, although there are a number of methods for utilising repeated measures in prediction models [29], most studies ignored this data structure. Studies should calculate and report sample size (and number of outcome events) and adequately assess model performance e both which are often poorly reported. We recommend:

- Develop MOEWSs that utilise repeated measure and report the number of observation sets used in the analysis [29].
- Use appropriately designed studies that are protocol-driven and informed by sample size calculations to mitigate against model over fitting and precise estimation of model performance [20,21].
- Include known predictors related to the outcome and use best practice statistical methods to determine what predictors to include and their relationship with the outcome, that is, nonlinearity in continuous predictors [61].
- Report the full model (all regression coefficients and intercept) or provide a link to code to run the model and allow external validation by independent researchers and potential downstream implementation [16,17].

#### 4.4.3 Consider missing data at each stage of model development

The methods for handling missing data in external validation studies should mimic how missing data is to be handled when using the model in clinical practice. Therefore, if missing values are permitted during implementation, then accounting for missing values (eg, imputing, use of sub models) should be evaluated during model validation. Missing indicator methods have been proposed to handle informative missingness but should be used with caution [23,62]. In the context of EWSs with repeated measurements over time, handling missing values is further complicated by the structure of the data with frequent missing predictors and varying missing data mechanisms. We recommend.

- Acknowledge missing data and report the amount of missingness for each predictor and outcome, along with any plausible assumptions on the missing data mechanisms [16,17,25,26,28].
- Describe how missing data were handled during model development and validation. It is recommended that CCA be avoided due to possible biases it can lead to, and it is good practice to use all available data [16,17].
- Consider how and whether missing values should be handled in the implementation of model, determine the best methods for accounting for missing data in real-time, and evaluate the model using these methods. Evaluate this approach during model validation [23,24].

## 5 Conclusion

Studies describing the development or validation of maternal EWSs were characteristics by poor methodological quality and were rated at high risk of bias that strongly suggests that published scores could perform poorly in clinical practice. Learning from the findings outlined in this study, we provide researchers with recommendations to support future development of robust reliable scores.

## Supplementary Material

**Supplementary data**

Supplementary data related to this article can be found at https://doi.org/10.1016/j.jclinepi.2025.111833.

Supplementary

## Figures and Tables

**Table 1:** Summary of the Irish and Scottish MEWS

Articles	Name of score	Country	Target population	Predictors
An Roinne Slante Department of Health, 2019 ([10,59])^a^	Irish MEWS	Ireland	Includes: women with a confirmed clinical pregnancy and for up to 42 d in the postnatal period, irrespective of age or reason for presentation. Excludes: women in labor, high dependency, recovery, and critical care settings	SBP, DBP, heart rate, respiratory rate, oxygen saturation, temperature, urine output, neuro response, and/or pain
Health Improvement Scotland, 2021 [11]^a^	Scottish MEWS	Scotland	Includes: women with a confirmed clinical pregnancy and up to 42 d postpartum. Excludes: women in labor.	SBP, DBP, heart rate, respiratory rate, oxygen saturation, temperature, urine output, neuro response, and/or looks unwell

MEWS, Maternal Early Warning System; SBP, Systolic blood pressure; DBP, Diastolic blood pressure.

a Scores were developed using clinical consensus and are not from a published study but are important to include as the first national scores implemented in maternity.

**Table 2:** Summary of the studies that developed an MOEWS

Model/Article	Country	Study design	Study period	Sample size	Model type	Outcome
Model developed using statistical methods (based on data)						
Escobar, 2020 [40]^a^	USA	Retrospective cohort	2010-2017	176,731	Logistic	Composite of maternal death, ICU admission and maternal morbidities.
Gorthi, 2009 [45]^c^	India	Simulated	NA	Not reported	Decision trees	Risk of maternal morbidity (not specified)
Raza, 2022 [46]^c^	Bangladesh	Unclear	Not reported	1218	Deep learning-based model	Risk of maternal morbidity (not specified)
Modified CEMACH MOEWS, Ryan, 2017^c^ [43]	Canada	Retrospective case-control cohort	2000–2011	184	Logistic	Admission to ICU for >24 hours during in-hospital stay
Developed using clinical consensus						
Modified CEMACH MOEWS, Hannola, 2021^a^ [47]	Finland	NA	NA	NA	NA	Composite of maternal morbidity
MEWT, Shields, 2016^b^ [42]	USA	NA	NA	NA	NA	Composite of maternal morbidity
MEWC, Mhyre, 2014^b^ [41]	USA	NA	NA	NA	NA	Maternal deterioration
Modified MEWC, Ibáñez-Lorente, 2021 [44]^a^	Spain	NA	NA	NA	NA	Composite of ICU admission and maternal morbidity

CEMACH MOEWS, confidential enquiry into maternal and child health modified obstetric early warning score; MEWT, maternal early warning trigger; MEWC, maternal early warning criteria; ICU, intensive care unit.

a Developed and externally validated in the same study.

b Externally validated.

c Developed only without external validation.

**Table 3:** Summary of the studies that validated an MOEWS

Study	Score(s) validated	Data sources	Country	Years of data	Sample size	Primary outcome
Development and external validation studies						
Escobar, 2020 [40]	Escobar, 2020	Retrospective cohort or dataset	USA	2017–2018	41,657	Composite of ICU admission, maternal mortality, and maternal morbidity
Hannola, 2021 [47]	Modified CEMACH MOEWS	Prospective cohort	Finland	2016–2018	828	Composite of maternal morbidity
Ibáñez Lorente, 2021 [44]	Modified MEWC	Prospective cohort	Spain	2018–2018	1166	Composite including ICU admission
Ryan, 2017 [43]	CEMACH MOEWS	Case-control study	Canada	2000–2011	184	ICU admission
Shields, 2016 [42]	MEWT	Prospective cohort	USA	2014–2015	12,611	Composite of maternal morbidity
External validation only studies						
Arnolds, 2019 [48]	MEWC	Retrospective cohort or dataset	USA	2016–2016	400	Composite of ICU admission, maternal mortality, and maternal morbidity
Arnolds, 2022 [49]	CEMACH MOEWS, MEWC, MEWT	Retrospective cohort or dataset	USA	2008–2018	19,611	Composite of ICU admission and maternal mortality
Blumenthal, 2019 [50]	CEMACH MOEWS, MEWC, MEWT	Retrospective cohort/dataset	USA	2016–2016	202	Composite of ICU admission, maternal mortality, and maternal morbidity
Blumenthal, 2021 [51]	MEWT	Retrospective cohort or dataset	USA	Not reported	204	Composite of ICU admission and maternal morbidity
Hedriana, 2016 [54]	MEWT	Case-control study	USA	2012–2013	54,429	ICU admission
Kern-Goldberger, 2022 [55]	CEMACH MOEWS, MEWC, MEWT	Retrospective cohort or dataset	USA	2018–2018	14,597	Composite of maternal morbidity
Rathore, 2018 [56]	ONEWS	Prospective cohort	India	2013–2015	500	Composite of ICU admission, maternal mortality, maternal morbidity
Singh, 2012 [52]	CEMACH MOEWS	Retrospective cohort or dataset	England	Not reported	676	Composite of ICU admission, maternal mortality, and maternal morbidity
Singh, 2016 [53]	CEMACH MOEWS	Prospective cohort	India	2012–2014	1065	Composite of maternal morbidity
Valent, 2017 [57]	CEMACH MOEWS	Retrospective cohort or dataset	USA	2012–2013	123	Composite of ICU admission and maternal morbidity

CEMACH MOEWS, confidential enquiry into maternal and child health modified obstetric early warning score; MEWT, maternal early warning trigger; MEWC, maternal early warning criteria; ICU, intensive care unit; ONEWS, Obstetric National Early Warning System.

## Data Availability

I have shared the DOI of my data in the manuscript.

## What is new?

**Key findings**

- Despite their widespread use in routine care, most modified obstetric early warning scores (MOEWSs) were not developed using recommended statistical methods
- Where MOEWSs were developed using datadriven approaches, they failed to report performance metrics and address key statistical challenges, such as handling repeated measures and managing missing data

**What this adds to what was known?**

- We show that most MOEWSs studies fail to adhere to recommended guidelines for clinical prediction model (CPM) development and validation.
- Our findings highlight the limitations of current MOEWS methodology, particularly in the handling of repeated measures and missing data

**What is the implication and what should change now?**

- Develop MOEWSs which exploit the repeated measures nature of the data
- Consider approaches to handle missing values for the real-time MOEWS implementation and evaluate the MOEWS performance using these approaches

## Plain Language Summary

Background: Pregnant women can become very ill very quickly and, if they do not receive rapid treatment, they can become seriously ill or even die. Tools called early warning scores (EWS) help doctors and midwives predict which women are likely to become unwell.

Aim: The aim of this study was to examine how existing tools were created to identify women who are more likely to have a life-threatening illness during pregnancy or shortly after birth.

Methods: We gathered all existing tools used to identify which pregnant women were at risk of illness. We gathered all the studies that tested how well a tool identified these women. We reviewed how these tools were developed and tested, for example, the patient information they used or how well they reported the results.

Results: We found 20 studies. Of these, 10 studies created tools (development studies) and 10 studies investigated the performance of these tools (validation studies). Most studies created simple tools that did not use robust scientific methodology and ignored how these tools would be used in practice such as when faced with missing information or when used repeatedly over pregnancy. The studies that tested the tools only examined simple tools and ignored the few more complicated tools that existed so we do not know how they would perform in new settings. Nearly all the studies were rated at high risk of bias.

Conclusion: There are a number of EWS that have been developed, but most did not use robust scientific methods. Future research needs to focus on robustly developed EWS, including how to deal with missing information, repeated measurements, and how well these models, work for different groups of women.

## Data sharing statement

All data extracted and the risk of bias for each study can be accessed online (DOI: 10.17605/OSF.IO/3FDSK)
