# Supplementary material for: Maternal early warning scores shown to be methodologically weak and at high risk of bias
Source: J Clin Epidemiol. Author manuscript; Available in PMC 2026 Mar 28. (PMC7618941; doi:10.1016/j.jclinepi.2025.111833)
Supplement: Supplementary [file EMS212877-supplement-Supplementary.docx]

# Supplementary Tables

Table A‑1: Summary of Open Science indicators for all studies

| **Study** | **Article open access** | **Data sharing agreement** | **Data open source** | **Methods reproducible** | **Protocol referenced** | **Registration number listed** | **Reporting guidelines mentioned** |
| --- | --- | --- | --- | --- | --- | --- | --- |
| **Development studies including development and validation studies** | | | | | | | |
| Irish MEWS, 2014 | Yes | NA | NA | Yes partially | No | No | No |
| Scottish MEWS, 2021 | Yes | NA | NA | No | No | No | No |
| Escobar, 2020 | No | No | No | Yes partially | No | No | No |
| Gorthi, 2009 | No | No | No | No | No | No | No |
| Hannola, 2021 | Yes | No | No | Yes partially | No | No | No |
| Ibanez Lorente, 2021 | No | Yes | Yes | Yes completely | No | No | Yes |
| Mhyre, 2014 | No | NA | NA | Yes partially | No | No | No |
| Raza, 2022 | Yes | Yes | Yes | No | No | No | No |
| Ryan, 2017 | Yes | No | No | Yes partially | No | No | Yes |
| Shields, 2016 | No | No | No | Yes partially | No | No | No |
| **External validation only studies** | | | | | | | |
| Arnolds, 2022 | Yes | Yes | No | Yes partially | No | No | No |
| Arnolds, 2019 | No | No | No | Yes partially | No | No | No |
| Blumenthal, 2019 | No | No | No | Yes partially | No | No | No |
| Blumenthal, 2021 | No | No | No | Yes partially | No | No | No |
| Hedriana, 2016 | No | No | No | Yes partially | No | No | No |
| Kern-Goldberger, 2022 | No | No | No | Yes completely | No | No | No |
| Rathore, 2018 | No | No | No | Yes partially | No | No | No |
| Singh, 2016 | No | No | No | Yes partially | No | No | No |
| Singh, 2012 | Yes | No | No | No | No | No | No |
| Valent, 2017 | No | No | No | Yes completely | No | No | No |

Table A‑2: Summary of predictors and which studies included them in their developed scores or models

| **Predictor** | **n** | **Irish MEWS** | **Scottish MEWS** | **MEWC, Mhyre** | **Modified MEWC, Ibanez-Lorente** | **MEWT, Shields, 2016** | **Modified CEMACH MOEWS Hannola, 2021** | **Escobar, 2020** | **Gorthi, 2009** | **Raza, 2022** | **Ryan, 2017** |
| --- | --- | --- | --- | --- | --- | --- | --- | --- | --- | --- | --- |
| SBP | 10 | Yes | Yes | Yes | Yes | Yes | Yes | Yes | Yes | Yes | Yes |
| DBP | 8 | Yes | Yes | Yes | Yes | Yes | Yes | No | Yes | Yes | No |
| Heart Rate | 8 | Yes | Yes | Yes | Yes | Yes | Yes | No | No | Yes | Yes |
| Temperature | 7 | Yes | Yes | No | No | Yes | Yes | Yes | No | Yes | Yes |
| Respiratory Rate | 7 | Yes | Yes | Yes | Yes | Yes | Yes | No | No | No | Yes |
| Oxygen saturation | 6 | Yes | Yes | Yes | Yes | Yes | Yes | No | No | No | No |
| Urine Output | 4 | Yes | Yes | Yes | Yes | No | No | No | No | No | No |
| Maternal Age | 3 | No | No | No | No | No | No | Yes | Yes | Yes | No |
| Neuro Response | 2 | Yes | Yes | No | No | No | No | No | No | No | No |
| Pain | 2 | Yes | No | No | No | No | Yes | No | No | No | No |
| Amniotic Fluid | 2 | No | No | No | No | No | No | No | Yes | No | No |
| Gestational Age | 2 | No | No | No | No | No | No | Yes | Yes | No | No |
| Looks Unwell | 1 | No | Yes | No | No | No | No | No | No | No | No |
| Accelerations | 1 | No | No | No | No | No | No | No | Yes | No | No |
| Admission Severity Score | 1 | No | No | No | No | No | No | Yes | No | No | No |
| Albuminuria | 1 | No | No | No | No | No | No | No | Yes | No | No |
| Basal Fetal Rate | 1 | No | No | No | No | No | No | No | Yes | No | No |
| Blood Glucose Level | 1 | No | No | No | No | No | No | No | No | Yes | No |
| Bmi Within 30 Days Of Admission | 1 | No | No | No | No | No | No | Yes | No | No | No |
| Cervical Dilation (Cm) | 1 | No | No | No | No | No | No | Yes | No | No | No |
| Comorbidity Score At Admission | 1 | No | No | No | No | No | No | Yes | No | No | No |
| Decelerations | 1 | No | No | No | No | No | No | No | Yes | No | No |
| Delivered (Y/N) | 1 | No | No | No | No | No | No | Yes | No | No | No |
| Diabetes Mellitus Indicator | 1 | No | No | No | No | No | No | Yes | No | No | No |
| Fever (Time With Temp >= 100.4 Farrenheightin H) | 1 | No | No | No | No | No | No | Yes | No | No | No |
| Fetal Movement | 1 | No | No | No | No | No | No | No | Yes | No | No |
| Gestational Diabetes | 1 | No | No | No | No | No | No | Yes | No | No | No |
| Gravidity | 1 | No | No | No | No | No | No | No | Yes | No | No |
| Growth Of Fetus | 1 | No | No | No | No | No | No | No | Yes | No | No |
| Heart Rate | 1 | No | No | No | No | Yes | No | No | No | No | No |
| Height | 1 | No | No | No | No | No | No | No | Yes | No | No |
| Hourly Severity Score In Previous 24 Hours | 1 | No | No | No | No | No | No | Yes | No | No | No |
| Hyperglycemia | 1 | No | No | No | No | No | No | No | Yes | No | No |
| Indicator For Dilation Missingness | 1 | No | No | No | No | No | No | Yes | No | No | No |
| Indicator For Station Missingness | 1 | No | No | No | No | No | No | Yes | No | No | No |
| Lochia | 1 | No | No | No | No | No | No | No | No | No | No |
| Maximum Temperature | 1 | No | No | No | No | No | No | No | No | No | Yes |
| Metorragia | 1 | No | No | No | No | No | No | No | Yes | No | No |
| Number Of Children Including Current Pregnancy | 1 | No | No | No | No | No | No | Yes | No | No | No |
| Placental Localization | 1 | No | No | No | No | No | No | No | Yes | No | No |
| Rupture Of Membranes (Elapsed Time Since) | 1 | No | No | No | No | No | No | Yes | No | No | No |
| Seizures | 1 | No | No | No | No | No | No | No | Yes | No | No |
| Severity Score Of >= 75 (time since) | 1 | No | No | No | No | No | No | Yes | No | No | No |
| Severity Score Of >= 75 (time with) | 1 | No | No | No | No | No | No | Yes | No | No | No |
| Umbilical Doppler | 1 | No | No | No | No | No | No | No | Yes | No | No |
| Uterine Contractions | 1 | No | No | No | No | No | No | No | Yes | No | No |
| Variability | 1 | No | No | No | No | No | No | No | Yes | No | No |
| Weight | 1 | No | No | No | No | No | No | No | Yes | No | No |
| Weight Gain | 1 | No | No | No | No | No | No | No | Yes | No | No |

Table A‑3: Details of the methods used by the four studies that developed a maternal early warning score using a statistical approach.

| **Article** | **Modelling approach** | **Variable selection approach** | **Multiple observation sets per patient and methods for handling** | **Handling of continuous predictors described** | **Full model described** | **Were risk groups created** | **Simplified model created** |
| --- | --- | --- | --- | --- | --- | --- | --- |
| Escobar, 2020 | Logistic | LASSO, gradient boosting, estimation of relative contribution, and clinical judgment | Yes: analysed at individual hospital stay, static and hourly. Number of observation sets not provided. | Yes | Partially: -2.39Delivered -0.12GA + 0.04ComorbidityScore +0.02HourlySeverityScore +0.03SBP +0.02AdmissionSeverityScore +0.05SeverityScoreMoreThan75(time with) -0.005ROM + 0.03MaternalAge -0.30StationCharted +0.20DilationCharted +0.17Temperature +0.24GestationalDiabetes -0.07Dilation + 1.74ShockIndex +0.14DiabetesMellitus -0.10Parity -0.005SeverityScoreMoreThan75(time since) +0.08Fever(time with) +0.009BMI | No | No |
| Gorthi, 2009 | Decision trees | Other - Literature survey of relevant parameters used in routine care | Unclear | No | No | No | No |
| Raza, 2022 | Deep learning-based model | Other - All predictors available in dataset | Unclear | No | No (not applicable) | No | No |
| Ryan, 2017 | Logistic | Stepwise | Yes: Included both highest and lowest value of vital signs. Number of observation sets not provided. | No | Partially – 1.14maximum temperature + 0.06HeartRate + 0.05SBP + 0.22RespiratoryRate | No | No |
| SBP Systolic Blood Pressure; BMI Body Mass Index; ROM Rupture of Membranes | | | | | | | |

Table A‑4: Sample size reported for the four papers reporting the development of a maternal early warning score using statistical methhods.

| **Article** | **Sample size** | **Outcome** | **Number of events (Units = % or n) ^1^** |
| --- | --- | --- | --- |
| **Model developed using statistical methods (based on data)** | | | |
| Escobar, 2020 | 176,731 | Composite of maternal death, ICU admission, maternal morbidities (eclampsia, severe preeclampsia with elevated severity of illness, haemorrhage, emboli, major deterioration without ICU transfer, uterine rupture) and neonatal outcomes (fetal death, neonatal hypoxic ischemic encephalopathy, neonatal acidosis) | Maternal death (0.01%), Composite (1.33%), more than 2 (0.25%)  Morbidites: AP haemorrhage (0.04%), AP pulmonary embolus (0.004%), AP severe preeclampsia (0.42%), AP eclampsia (0.003%), AP transfer to ICU for cause (0.01%), AP LAPS2 of more than 110 (0.01%), Intrauterine fetal demise (0.02%), neonatal acidosis (0.32%), Hypoxic ischemic encephalopathy (0.12%), Uterine Rupture (0.09%), PP haemorrhage (0.31%), PP pulmonary embolus (0.009%), PP severe preeclampsia (0.03%), PP eclampsia (0.002%), PP transfer to ICU for cause (0.20%), PP LAPS2 of more than 120 (0.10%), PP death (0.01%), Aggregated severe preeclampsia (0.46%), fetal or neonatal (0.38%), |
| Gorthi, 2009 | Not reported | Categories of risk complication | Not reported |
| Raza, 2022 | 1,218 | Categories of risk complication | Not reported |
| Ryan, 2017 | 184 | Admission to ICU for >24 hours during in-hospital stay | ICU admission (N=46) |
| **Developed using clinical consensus** | | | |
| Hannola, 2021 | NA | Composite of maternal morbidity (preeclampsia, infection including sepsis or PP haemorrhage) | NA |
| Shields, 2016 | NA | Composite of maternal morbidity (infection/sepsis, pulmonary, severe pre-eclampsia-hypertension and severe haemorrhage) | NA |
| Mhyre, 2014 | NA | Maternal deterioration |  |
| Ibáñez -Lorente , 2021 | NA | Composite of ICU admission, surgery within two hours of delivery and length of stay more than seven days | NA |
| ^1^Number of events at observation level were not presented by any study  AP Antepartum, PP Postpartum | | | |

Table A‑5: Predictor variables considered as candidates and included in the final model, reported for the papers reporting the development of a maternal early warning score

| **Article** | **Number of candidate predictors** | **Number of predictors in final model** | **Predictors in final model** |
| --- | --- | --- | --- |
| **Model developed using statistical methods (based on data)** | | | |
| Escobar, 2020 | NA | 35 | Delivered (y/n), Gestational age (week), Comorbidity score at admission, Hourly severity score in previous 24 hours, SBP (lowest within the hour), Admission severity score, Severity score of >= 75, Rupture of Membranes (elapsed time since), Maternal age (years at admission), indicator for station missingness, indicator for dilation missingness, Temperature (highest within the hour), Gestational Diabetes, Cervical dilation (cm), Shock Index (highest heart rate/lowest SBP), Diabetes mellitus indicator, Number of children including current pregnancy, Severity score of >=75, Fever (time with temp >= 100.4 Fahrenheit), BMI within 30 days of admission |
| Gorthi, 2009 | 31 | 22 | Maternal Age, Gravidity, Gestational Age, Height, Weight, Weight gain, Blood Pressure, Metorragia, Uterine Contractions, Amniotic Fluid Loss, Seizures, Albuminuria, Hyperglycemia, Basal fetal heart rate, Variability, Accelerations, Decelerations, Fetal Movement, Placental Localization, Growth of Fetus, Umbilical Doppler |
| Raza, 2022 | 6 | 6 | Maternal age, SBP, DBP, Blood glucose level, Temperature, Heart Rate |
| Ryan, 2017 | 8 | 4 | Maximum temperature, heart rate, SBP, respiratory rate |
| **Developed using clinical consensus** | | | |
| Hannola, 2021 | Not reported | NA | SBP, DBP, heart rate, respiratory rate, oxygen saturation, temperature, pain score |
| Shields, 2016 | Not reported | NA | SBP, DBP, heart rate, respiratory rate, oxygen saturation, temperature, altered mental status, mean arterial pressure, disproportionate pain, fetal heart rate |
| Mhyre, 2014 | Not reported | NA | SBP, DBP, heart rate, respiratory rate, oxygen saturation, urine output, maternal agitation, confusion or unresponsiveness and patient with preeclampsia reporting a non-remitting headache or shortness of breath |
| Ibáñez -Lorente , 2021 | Not reported | NA | SBP, DBP, heart rate, respiratory rate, oxygen saturation, urine output, uterine involution measurement, bleeding greater than 500ml |
| SBP Systolic blood pressure, DBP diastolic blood pressure, BMI Body Mass Index | | | |

Table A‑6: Summary of the approach to missing data for the four studies that developed a MOEWS using data

| **Article** | **Missing data during development discussed** | **Amount of missing data presented** | **Missing data assumption** | **Handling of missing data** | **Effect of missing data at implementation discussed** |
| --- | --- | --- | --- | --- | --- |
| Escobar, 2020 | Reported | Missing data reported by outcome status for predictors | Unclear | LOCF, imputed to normal, missing indicators, and trajectories | Not discussed |
| Gorthi, 2009 | Not reported | Not reported | Not reported | Not reported | Not discussed |
| Raza, 2022 | Not reported | Not reported | Not reported | Not reported | Not discussed |
| Ryan, 2017 | Reported | Missing data reported by outcome status for predictors. | MAR | Complete cases | Not discussed |
| MAR Missing at Random, LOCF Last observation carried forward | | | | | |

Table A‑7: Assessment of apparent performance in the four studies developing a maternal early warning score using a data-driven approach

| **Study** | **Apparent performance assessed** | **Discrimination** | **Calibration** | **Sensitivity** | **Specificity** | **PPV** | **NPV** |
| --- | --- | --- | --- | --- | --- | --- | --- |
| Escobar, 2020 | No | Not reported | Not reported | Not reported | Not reported | Not reported | Not reported |
| Gorthi, 2009 | No | Not reported | Not reported | Not reported | Not reported | Not reported | Not reported |
| Raza, 2022 | No | Not reported | Not reported | Not reported | Not reported | Not reported | Not reported |
| Ryan, 2017 | Yes | Not reported | Not reported | Reported | Reported | Not reported | Reported |
| PPV Positive Performance Value  NPV Negative Performance Value | | | | | | | |

Table A‑8: Reporting of the internal validation by the studies that developed a MOEWS on data

| **Study** | **IV assessed** | **IV method** | **Discrimination** | **Calibration** | **Sensitivity** | **Specificity** | **PPV** | **NPV** |
| --- | --- | --- | --- | --- | --- | --- | --- | --- |
| Escobar, 2020 | Yes | Split sample | Not reported | Not reported | Not reported | Not reported | Not reported | Not reported |
| Gorthi, 2009 | Yes | Cross-validation | Not reported | Not reported | Not reported | Not reported | Not reported | Not reported |
| Raza, 2022 | Yes | Split sample | Not reported | Not reported | Not reported | Not reported | Not reported | Not reported |
| Ryan, 2017 | No | None | Not reported | Not reported | Not reported | Not reported | Not reported | Not reported |
| IV Internal validation  PPV Positive Performance Value  NPV Negative Performance Value | | | | | | | | |

Table A‑9: Summary of included validation studies with population and primary outcome

| **Study** | **Score(s) validated** | **Data source** | **Population** | **Primary outcome** |
| --- | --- | --- | --- | --- |
| Arnolds, 2019 | MEWC | Retrospective cohort/dataset, | In-hospital admission for delivery after 23 weeks gestation and all observations recorded until the second stage of labour excluding the anaesthetic record. Intrapartum. | Composite of ICU admission, maternal mortality and maternal morbidity (haemorrhage, pre-eclampsia with severe features, suspected infection, pulmonary embolus, cerebral venous sinus thrombosis, intracranial bleed, acute asthma, status epilepticus, diabetic ketoacidosis, myocardial infarction, pulmonary oedema, anaesthetic complication) |
| Arnolds, 2022 | CEMACH MOEWS, MEWC, MEWT | Retrospective cohort/dataset, | Women ≥18 years admitted to a hospital ward after transfer from labour and delivery. The cohort includes both postpartum patients as well as patients who were initially admitted to labour and delivery prior to transfer to the antepartum ward. | Composite of ICU admission and maternal mortality within 24 hours of a ward observation |
| Blumenthal, 2019 | CEMACH MOEWS, MEWC, MEWT | Retrospective cohort/dataset, USA | In-hospital admission for delivery. Intrapartum to early post-partum | Composite of ICU admission, maternal mortality or morbidity (acute myocardial infarction, acute renal failure, adult respiratory distress syndrome, amniotic fluid embolism, aneurysm, cardiac arrest/ventricular fibrillation, disseminated intravascular coagulation, eclampsia, heart failure during procedure or surgery, internal injuries of thorax or abdomen or pelvis, intracranial injuries, puerperal cerebrovascular disorder, pulmonary oedema, severe anaesthesia complications, sepsis, shock, sickle cell anaemia with crisis, blood transfusion, cardiac monitoring, conversion of cardiac rhythm, hysterectomy, operations on heart or pericardium, temporary tracheostomy, ventilation) |
| Blumenthal, 2021 | MEWT | Retrospective cohort/dataset, USA | In-hospital admission for delivery. Intrapartum to early post-partum. | Composite of ICU admission, prolonged postpartum length of stay, maternal morbidity (sepsis/severe sepsis, cardiopulmonary, hypertension, haemorrhage) |
| Escobar, 2020 | Escobar, 2020 | Retrospective cohort | Admitted for delivery, GA more than 22 weeks; fetal heart rate active on admission; delivery (live birth or fetal loss) occurred after admission. Antepartum and Postpartum | Composite of ICU admission, maternal mortality, maternal morbidity (eclampsia, severe preeclampsia with elevated severity of illness, haemorrhage, emboli, major deterioration without ICU transfer, uterine rupture) and neonatal outcomes (fetal death, neonatal hypoxic ischemic encephalopathy, neonatal acidosis) |
| Hannola, 2021 | Obstetric EWS (An adapted version of CEMACH MOEWS) and the CEMACH MOEWS | Prospective cohort/dataset | Women considered to have an increased risk for morbidity after birth (BMI > 35 kg/m^2^, PPH > 1500 g, pre-eclampsia, clinical judgement, chorioamnionitis during birth or type 1 diabetes). Early postpartum after transfer from labour room or caesarean section recovery room | Composite of maternal morbidity (preeclampsia, infection/sepsis, post-partum haemorrhage) |
| Hedriana, 2016 | MEWT | Retrospective case-control study, USA. | Cases were women at term or preterm requiring obstetric triage (hypertension, vaginal bleeding, abdominal pain, ruptured membranes, fever, gastrointestinal symptoms, etc.) who were transferred to ICU from a maternal ward (antepartum, intrapartum and postpartum) Exclusion criteria was direct admission to ICU from the emergency department.  Control group were women admitted to the maternity units after triage with normal delivery outcome over a 24-hour period. | ICU admission at any point during hospital admission after transfer from a maternal ward |
| Ibáñez -Lorente , 2021 | Modified MEWC | Prospective cohort/dataset | Pregnant women admitted to hospital for delivery. Early post-partum. | Composite of ICU admission, surgery within two hours of delivery and length of stay more than seven days |
| Kern-Goldberger, 2022 | MOEWS, MEWC, MEWT | Retrospective cohort/dataset | Pregnant women at any gestational age, 18+ who were admitted for delivery. Intrapartum and immediate postpartum | Composite of maternal morbidity (haemorrhage, infection, acute cardiovascular disease, acute pulmonary disease) |
| Rathore, 2018 | ONEWS | Prospective cohort/dataset | Pregnant women or within 42 days of end of pregnancy who had any one or more of: high-risk pregnant woman admitted from obstetrics and gynaecology emergency, had undergone surgery within the last 24h, and was already admitted in obstetrics ward and became sick. Women admitted directly to the ICU were excluded. | Composite of ICU admission, maternal mortality and maternal morbidity (haemorrhage, pre-eclampsia/eclampsia, renal dysfunction, liver dysfunction, cardiac arrest, pulmonary oedema, acute respiratory dysfunction, cerebrovascular event, septicaemic shock, massive pulmonary embolism) |
| Ryan, 2017 | CEMACH MEOWS | Retrospective case-control study, Canada, | Pregnant or recently pregnant (<6 weeks after the end of the pregnancy, irrespective of gestational age at the end of the pregnancy) admitted women who subsequently required admission to the ICU for >24 hours. Antepartum and immediate post-partum. | Admission to ICU for >24 hours during in-hospital stay |
| Shields, 2016 | Shields, MEWT, 2016 | Prospective, USA | Pregnant women admitted to hospital maternity centre. Any stage of pregnancy. | Composite of maternal morbidity (infection/sepsis, pulmonary, severe pre-eclampsia-hypertension and severe haemorrhage) |
| Singh, 2012 | CEMACH MOEWS | Retrospective cohort/dataset, England | Women between 20 weeks gestation and 6 weeks postpartum who were admitted as an inpatient to the maternity unit. Any stage of pregnancy | Composite of ICU admission maternal mortality and maternal morbidity (haemorrhage, pre-eclampsia, suspected infection, anaesthetic complication, acute asthma) occurring within 30 days of in-hospital admission |
| Singh, 2016 | CEMACH MOEWS | Prospective cohort | Pregnant women in labour beyond 28 weeks gestation and up to 6 weeks postpartum | Composite of maternal morbidity (hypertensive disorder of pregnancy, eclampsia, obstetric haemorrhage, suspected infection, pulmonary oedema, shock, gestational diabetes, diabetic ketoacidosis, intracranial bleed, acute asthma, status epilepticus, neonatal outcomes) |
| Valent, 2017 | CEMACH MOEWS | Retrospective cohort/dataset, | Pregnant women admitted to hospital and diagnosed with acute pyelonephritis during antepartum period | Composite of ICU admission or maternal morbidity (pulmonary injury, blood transfusion, sepsis) |

Table A‑10: Summary of sample size of validation studies

| **Article** | **Sample size** | **Outcome (events, Units = % or n)** | **Number of events presented at observation level** |
| --- | --- | --- | --- |
| Escobar, 2020 | 41657 | Total (N= 638, 1.53%), Antepartum hemorrhage (0.04), AP pulmonary embolus (0.01), Antepartum severe preeclampsia (0.29), Antepartum eclampsia (0.005), Antepartum transfer to ICU for cause (0.01), Antepartums LAPS2 of more than 110 (0.04), Intrauterine fetal demise (0.06), neonatal acidosis (0.43), Hypoxic ischemic encephalopathy (0.16), Uterine Rupture (0.12), PP haemorrhage (0.35), PP pulmonary embolus (0.005), PP severe preeclampsia (0.02), PP eclampsia <0.001, PP transfer to ICU for cause (0.30), PP LAPS2 of more than 120 (0.16), PP death (0.01), Aggregated severe preeclampsia (0.31), fetal or neonatal (0.53), maternal (1.04), Composite (1.53), more than 2 (0.35) | No |
| Hannola, 2021 | 828 | Pre-eclampsia (n=65), PPH (n=112), Infection (n=33) | No |
| Ryan, 2017 | 184 | 46 ICU admissions | No |
| Shields, 2016 | 12611 | ICU admission (n=47) and/or composite of maternal morbidity (n=1); sepsis (n=1.3/1000); cardiovascular dysfunction (not reported), severe preeclampsia-hypertension (not reported), and severe haemorrhage (n=0.03)) | No |
| Arnolds, 2022 | 19611 | 43 ICU admissions or deaths, 3 deaths | No |
| Arnolds, 2019 | 400 | 99/400 (25%) total. Obstetric hemorrhage (48, 12%), preeclampsia with severe features (23, 6%), suspected infection (37, 9%), pulmonary embolus (5, 1%), cerebral venous sinus thrombosis (0), intracranial bleed (0), acute asthma (0), status epilepticus (0), diabetic ketoacidosis (1 <1%), myocardial infarction (0), pulmonary edema (5, 1%), anesthetic complication (1, <1%), any ICU admission (2 ,<1%), maternal mortality (1 <1%) | No |
| Blumenthal, 2021 | 204 | Composite of ICU admission, maternal morbidity in-hospital (2.06/100 deliveries) | No |
| Blumenthal, 2019 | 202 | Blood transfusion (62, 78.5), Pulmonary edema (12, 15.2), Hysterectomy (11, 3.9), Adult respiratory distress syndroms (8, 10.1), Disseminated intravascular coagulation (7, 8.9%), Acute renal failure (7, 8.9%), Eclampsia (5, 6.3%), Sepsis (4, 5.1%), Internal injuries of the thorax, abdomen, pelvis (4, 5.1), Cardiac monitoring (3, 3.8%), Shock (1, 1.3%), Myocardial infarction (1, 1.3%), Cardiac arrest (1, 1.3%), Obstetric haemorrhage (21, 26.6%), Prolonged postpartum length of stay (12, 15.2%), preeclampsia (10, 12.7%), ICU admission (9, 11.4%), Maternal mortality (1, 1.3%) | No |
| Hedriana, 2016 | 54429* | ICU admission (n=262) | No |
| Ibanez Lorente, 2021 | 1166 | ICU Admission (n=18, 1.5%), surgery (n=15, 1.3%), Length of stay >7 days (n=13, 1.1%) | No |
| Kern-Goldberger, 2022 | 14597 | 2451 at least one morbidity event (16.8%), 980 haemorrhage (6.7%), 1337 infection (9.2%), 362 Acute Cardiovascular Disease (2.5%), 275 Acute Pulmonary Disease (1.9%) | No |
| Rathore, 2018 | 500 | CAMO (n=159), haemorrhage (n=29), pre-eclampsia/eclampsia (n=96), renal dysfunction (n=13), liver dysfunction (n=14), cardiac arrest (n=7), pulmonary oedema (n=6), acute respiratory dysfunction (n=5), cerebrovascular event (n=1), septicaemic shock (n=2), massive pulmonary embolism (n=3), ICU admission and maternal death in-hospital | No |
| Singh, 2016 | 1065 | Hypertensive disorder of pregnancy (69%), eclampsia, obstetric haemorrhage, suspected infection, pulmonary oedema, shock, gestational diabetes, diabetic ketoacidosis, intracranial bleed, acute asthma, status epilepticus, neonatal outcomes | No |
| Singh, 2012 | 676 | Obstetric haemorrhage (43%), Pre-eclampsia (31%), Suspected Infection (20%), Anaesthetic complication (4%), Acute asthma (2%) | No |
| Valent, 2017 | 123* | Pulmonary injury (2), ICU admission, blood transfusion (7), or sepsis (8) | No |

Table A‑11: Summary of missing data in validation studies

| **Study** | **Missing data referred to** | **Proportion missing** | **Assumption** | **Approach** |
| --- | --- | --- | --- | --- |
| Arnolds, 2019 | No | Not reported | Not reported | Not reported |
| Arnolds, 2022 | No | Not reported | Not reported | Not reported |
| Blumenthal, 2019 | No | Not reported | Not reported | Not reported |
| Blumenthal, 2021 | No | Not reported | Not reported | Not reported |
| Escobar, 2020 | Yes | Not reported | Unclear | LOCF, imputed to normal, missing indicators, and trajectories |
| Hannola, 2021 | No | Not reported | Not reported | Not reported |
| Hedriana, 2016 | No | Not reported | Not reported | Not reported |
| Ibanez Lorente, 2021 | No | Not reported | Not reported | Not reported |
| Kern-Goldberger, 2022 | No | Not reported | Not reported | Not reported |
| Rathore, 2018 | No | Not reported | Not reported | Not reported |
| Ryan, 2017 | Yes | Missing data reported by outcome status for predictors. | Other: When MEOWS variables were missing, it was assumed that these observations were not recorded because they were not perceived to be abnormal and they would not have fallen into the 'red' or 'amber' trigger categories. | Use of complete cases |
| Shields, 2016 | No | Not reported | Not reported | Not reported |
| Singh, 2012 | Yes | Number of completed charts reported | Unclear | Filled in missing by reviewing all notes available |
| Singh, 2016 | No | Not reported | Not reported | Not reported |
| Valent, 2017 | No | Not reported | Not reported | Not reported |

Table A‑12: Reporting of the performance metrics of the validation studies

| **Study** | **Discrimination** | **Calibration** | **Sensitivity** | **Specificity** | **NPV** | **PPV** |
| --- | --- | --- | --- | --- | --- | --- |
| Arnolds, 2019 | No | No | Assessed | Assessed | Assessed | Assessed |
| Arnolds, 2022 | Yes | No | Assessed | Assessed | Assessed | Assessed |
| Blumenthal, 2019 | No | No | Assessed | Assessed | Not assessed | Not assessed |
| Blumenthal, 2021 | No | No | Assessed | Assessed | Not assessed | Not assessed |
| Escobar, 2020 | Yes | Yes | Assessed | Assessed | Not assessed | Assessed |
| Hannola, 2021 | No | No | Assessed | Assessed | Assessed | Assessed |
| Hedriana, 2016 | No | No | Assessed | Assessed | Assessed | Assessed |
| Ibanez Lorente, 2021 | No | No | Assessed | Assessed | Assessed | Assessed |
| Kern-Goldberger, 2022 | No | No | Not assessed | Assessed | Assessed | Assessed |
| Rathore, 2018 | Yes | No | Assessed | Assessed | Assessed | Assessed |
| Ryan, 2017 | No | No | Assessed | Assessed | Assessed | Assessed |
| Shields, 2016 | No | No | Assessed | Assessed | Assessed | Assessed |
| Singh, 2012 | No | No | Assessed | Assessed | Assessed | Assessed |
| Singh, 2016 | No | No | Assessed | Assessed | Not assessed | Not assessed |
| Valent, 2017 | Yes | No | Assessed | Assessed | Assessed | Assessed |

# Appendix B Search Strategies

The search strategy for each database (developed by an information specialist) comprised a combination of free-text terms and relevant controlled vocabulary headings (e.g. Mesh, EMTREE etc.) related to the intervention (early warning, trigger, prediction), outcomes of interest (maternal mortality, obstetric morbidity) and model design, development or validation (Appendix 1). Apart from the date limit mentioned above no other limits were applied to the search. MCJ screened the abstracts of all studies, with a second reviewer consulted as necessary (GSC or SG).

Database and platform: CINAHL (via EBSCOHost)

Search run on: 21 March 2023

1. TI ("Modified Early Obstetric Warning Score*" OR "Modified Early Obstetric Warning System*" OR "Maternal Early Recognition Criteria" OR "Modified Early Warning System*" OR "Maternal Early Warning Trigger*" OR "Maternal Early Obstetric Warning System*" OR "Irish Maternity Early Warning System*" OR "Obstetric Early Warning Score*" OR "Obstetric early warning system*" OR "Maternal Early Warning Criteria" OR "Modified Obstetric Early Warning Score*" OR "MOEWS" OR "MEWT" OR "MEWTs" OR "MEOWS" OR "MEWC" OR "MERC") OR AB ("Modified Early Obstetric Warning Score*" OR "Modified Early Obstetric Warning System*" OR "Maternal Early Recognition Criteria" OR "Modified Early Warning System*" OR "Maternal Early Warning Trigger*" OR "Maternal Early Obstetric Warning System*" OR "Irish Maternity Early Warning System*" OR "Obstetric Early Warning Score*" OR "Obstetric early warning system*" OR "Maternal Early Warning Criteria" OR "Modified Obstetric Early Warning Score*" OR "MOEWS" OR "MEWT" OR "MEWTs" OR "MEOWS" OR "MEWC" OR "MERC")

2. (MH "Early Warning Score") OR (MH "Early diagnosis") OR (MH "Severity of Illness Indices") OR (MH "Vital Signs") OR (MH "Decision Support Systems, Clinical")

3. TI (early N2 (warning or recognition or surveillance) N1 (trigger* or criteria or score* or system*)) OR AB (early N2 (warning or recognition or surveillance) N1 (trigger* or criteria or score* or system*))

4. S2 OR S3

5. TI (develop* OR design* OR creat* OR build* OR contruct* OR validat* OR evaluat*) OR AB (develop* OR design* OR creat* OR build* OR contruct* OR validat* OR evaluat*)

6. TI (prognos* N1 (modelling OR modeling OR model OR models OR predict* OR performance OR tools OR ability OR accuracy OR probability OR risk OR factor*)) OR AB (prognos* N1 (modelling OR modeling OR model OR models OR predict* OR performance OR tools OR ability OR accuracy OR probability OR risk OR factor*))

7. TI ("risk model*" OR "predict* the prognosis of" OR "predict* the risk of" OR "predict* the probability of" OR "candidate predictor*" OR "predictive clinical parameter*" OR "descriptive model*") OR AB ("risk model*" OR "predict* the prognosis of" OR "predict* the risk of" OR "predict* the probability of" OR "candidate predictor*" OR "predictive clinical parameter*" OR "descriptive model*")

8. TI (probability N1 (modelling or modeling or model or models)) OR AB (probability N1 (modelling or modeling or model or models))

9. TI (predict* N1 (modelling or modeling or model or models or tools or performance or ability or accuracy or probability or risk)) OR AB (predict* N1 (modelling or modeling or model or models or tools or performance or ability or accuracy or probability or risk))

10. TI ((prognos* or predict*) N1 (index or nomogram))

11. S6 OR S7 OR S8 OR S9 OR S10

12. (MH "Pregnancy") OR (MH "Maternal Mortality") OR (MH "Pregnancy Complications") OR (MH "Pregnancy outcomes") OR (MH "Labor Complications+") OR (MH "Pregnancy Complications, Infectious") OR (MH "Pregnancy-Induced Hypertension+") OR (MH "Uterine Rupture")

13. TI ((maternal OR maternity OR obstetric* OR perinatal OR pregnancy OR "antepartum" OR "ante-partum" OR "ante partum" OR delivery OR labour OR labor) N2 (morbidity OR death OR mortality OR "vital sign*" OR decompensation OR deteriorat* OR complication* OR infection* OR sepsis OR "ICU admission" OR "critical care" OR "intensive care" OR "high dependency unit*" OR "HDU" OR "HDUs" OR "critical level 2")) OR AB ((maternal OR maternity OR obstetric* OR perinatal OR pregnancy OR "antepartum" OR "ante-partum" OR "ante partum" OR delivery OR labour OR labor) N2 (morbidity OR death OR mortality OR "vital sign*" OR decompensation OR deteriorat* OR complication* OR infection* OR sepsis OR "ICU admission" OR "critical care" OR "intensive care" OR "high dependency unit*" OR "HDU" OR "HDUs" OR "critical level 2"))

14. TI (("post partum" OR "postpartum" OR "post-partum" OR "post natal" OR "postnatal" OR "post-natal" OR "post delivery" OR "postdelivery" OR "post-delivery") N2 (haemorrhage OR hemorrhage OR bleed* OR "blood loss")) OR AB (("post partum" OR "postpartum" OR "post-partum" OR "post natal" OR "postnatal" OR "post-natal" OR "post delivery" OR "postdelivery" OR "post-delivery") N2 (haemorrhage OR hemorrhage OR bleed* OR "blood loss"))

15. TI ("pre-eclampsia" OR eclampsia OR preeclampsia OR HELLP OR "acute fatty liver of pregnancy" OR "rupture of the uterus") OR AB ("pre-eclampsia" OR eclampsia OR preeclampsia OR HELLP OR "acute fatty liver of pregnancy" OR "rupture of the uterus")

16. TI ((uterus OR uterine) N1 (ruptur* or perforat*)) OR AB ((uterus OR uterine) N1 (ruptur* or perforat*))

17. S12 OR S13 OR S14 OR S15 OR S16

18. S4 OR S11

19. S5 AND S17 AND S18

20. S1 OR S19

21. PY 2000 OR PY 2001 OR PY 2002 OR PY 2003 OR PY 2004 OR PY 2005 OR PY 2006 OR PY 2007 OR PY 2008 OR PY 2009 OR PY 2010 OR PY 2011 OR PY 2012 OR PY 2013 OR PY 2014 OR PY 2015 OR PY 2016 OR PY 2017 OR PY 2018 OR PY 2019 OR PY 2020 OR PY 2021 OR PY 2022 OR PY 2023

22. S20 AND S21

RESULTS = 3266 references

Database and platform: Embase 1974 to present (via OVID)

Search run on: 21 March 2023

1. ("Modified Early Obstetric Warning Score$" or "Modified Early Obstetric Warning System$" or "Maternal Early Recognition Criteria" or "Modified Early Warning System$" or "Maternal Early Warning Trigger$" or "Maternal Early Obstetric Warning System$" or "Irish Maternity Early Warning System$" or "Obstetric Early Warning Score$" or "Obstetric early warning system$" or "Maternal Early Warning Criteria" or "Modified Obstetric Early Warning Score$" or "MOEWS" or "MEWT" or "MEWTs" or "MEOWS" or "MEWC" or "MERC").mp.

2. exp Early warning score/ or Scoring system/ or Vital sign/ or Disease severity assessment/

3. (early adj2 (warning or recognition or surveillance) adj1 (trigger* or criteria or score$ or system$)).ti,ab,kw.

4. or/2-3

5. (develop$ or design$ or creat$ or build$ or contruct$ or validat$ or evaluat$).ti,ab.

6. (prognos$ adj1 (modelling or modeling or model or models or predict$ or performance or tools or ability or accuracy or probability or risk or factor$)).ti,ab,kw.

7. ("risk model$" or "predict$ the prognosis of" or "predict$ the risk of" or "predict$ the probability of" or "candidate predictor$" or "predictive clinical parameter$" or "descriptive model$").ti,ab,kw.

8. (probability adj1 (modelling or modeling or model or models)).ti,ab,kw.

9. (predict$ adj1 (modelling or modeling or model or models or tools or performance or ability or accuracy or probability or risk)).ti,ab,kw.

10. ((prognos$ or predict$) adj1 (index or nomogram)).ti.

11. or/6-10

12. Maternal Death/ or Maternal Mortality/ or exp Pregnancy complication/ or exp Labor complication/ or Obstetric emergency/ or exp "Eclampsia and preeclampsia"/ or Obstetric delivery/ or Maternal morbidity/

13. ((maternal or maternity or obstetric$ or perinatal or pregnancy or "antepartum" or "ante-partum" or "ante partum" or delivery or labour or labor) adj2 (morbidity or death or mortality or "vital sign$" or decompensation or deteriorat$ or complication$ or infection$ or sepsis or "ICU admission" or "critical care" or "intensive care" or "high dependency unit$" or "HDU" or "HDUs" or "critical level 2")).ti,ab,kw.

14. (("post partum" or "postpartum" or "post-partum" or "post natal" or "postnatal" or "post-natal" or "post delivery" or "postdelivery" or "post-delivery") adj2 (haemorrhage or hemorrhage or bleed$ or "blood loss")).ti,ab,kw.

15. ("pre-eclampsia" or eclampsia or preeclampsia or HELLP or "acute fatty liver of pregnancy" or "rupture of the uterus").ti,ab,kw.

16. ((uterus or uterine) adj1 (ruptur$ or perforat$)).ti,ab,kw.

17. or/12-16

18. 4 or 11

19. 5 and 17 and 18

20. 1 or 19

21. conference abstract.pt.

22. conference abstract.st.

23. 21 or 22

24. 20 not 23

25. limit 24 to yr="2000-2023"

RESULTS = 5751 references

Database and platform: Medline (Ovid MEDLINE® Epub Ahead of Print, In-Process & Other Non-Indexed Citations, Ovid MEDLINE® Daily and Ovid MEDLINE®) 1946 to present

Search run on: 21 March 2023

1. ("Modified Early Obstetric Warning Score$" or "Modified Early Obstetric Warning System$" or "Maternal Early Recognition Criteria" or "Modified Early Warning System$" or "Maternal Early Warning Trigger$" or "Maternal Early Obstetric Warning System$" or "Irish Maternity Early Warning System$" or "Obstetric Early Warning Score$" or "Obstetric early warning system$" or "Maternal Early Warning Criteria" or "Modified Obstetric Early Warning Score$" or "MOEWS" or "MEWT" or "MEWTs" or "MEOWS" or "MEWC" or "MERC").mp.

2. Early Warning Score/ or Early diagnosis/ or Severity of Illness Index/ or Vital Signs/ or "Decision Support Systems, Clinical"/

3. (early adj2 (warning or recognition or surveillance) adj1 (trigger$ or criteria or score$ or system$)).ti,ab,kw.

4. or/2-3

5. (develop$ or design$ or creat$ or build$ or contruct$ or validat$ or evaluat$).ti,ab.

6. (prognos$ adj1 (modelling or modeling or model or models or predict$ or performance or tools or ability or accuracy or probability or risk or factor$)).ti,ab,kw.

7. ("risk model$" or "predict$ the prognosis of" or "predict$ the risk of" or "predict$ the probability of" or "candidate predictor$" or "predictive clinical parameter$" or "descriptive model$").ti,ab,kw.

8. (probability adj1 (modelling or modeling or model or models)).ti,ab,kw.

9. (predict$ adj1 (modelling or modeling or model or models or tools or performance or ability or accuracy or probability or risk)).ti,ab,kw.

10. ((prognos$ or predict$) adj1 (index or nomogram)).ti.

11. or/6-10

12. Pregnancy/ or Maternal Death/ or Maternal Mortality/ or Pregnancy Complications/ or Pregnancy outcome/ or exp Obstetric Labor Complications/ or exp Pregnancy Complications, Infectious/ or exp Hypertension, Pregnancy-Induced/

13. ((maternal or maternity or obstetric$ or perinatal or pregnancy or "antepartum" or "ante-partum" or "ante partum" or delivery or labour or labor) adj2 (morbidity or death or mortality or "vital sign$" or decompensation or deteriorat$ or complication$ or infection$ or sepsis or "ICU admission" or "critical care" or "intensive care" or "high dependency unit$" or "HDU" or "HDUs" or "critical level 2")).ti,ab,kw.

14. (("post partum" or "postpartum" or "post-partum" or "post natal" or "postnatal" or "post-natal" or "post delivery" or "postdelivery" or "post-delivery") adj2 (haemorrhage or hemorrhage or bleed$ or "blood loss")).ti,ab,kw.

15. ("pre-eclampsia" or eclampsia or preeclampsia or HELLP or "acute fatty liver of pregnancy" or "rupture of the uterus").ti,ab,kw.

16. ((uterus or uterine) adj1 (ruptur$ or perforat$)).ti,ab,kw.

17. or/12-16

18. 4 or 11

19. 5 and 17 and 18

20. 1 or 19

21. limit 20 to yr="2000-2023"

RESULTS = 7949 references

Database and platform: Web of Science Core Collection (via Clarivate at https://www.webofscience.com/wos/woscc/)

Edition: Science Citation Index Expanded (SCI-EXPANDED)-- 1900-present

Search run on: 21 March 2023

1. TS=("Modified Early Obstetric Warning Score*" OR "Modified Early Obstetric Warning System*" OR "Maternal Early Recognition Criteria" OR "Modified Early Warning System*" OR "Maternal Early Warning Trigger*" OR "Maternal Early Obstetric Warning System*" OR "Irish Maternity Early Warning System*" OR "Obstetric Early Warning Score*" OR "Obstetric early warning system*" OR "Maternal Early Warning Criteria" OR "Modified Obstetric Early Warning Score*" OR "MOEWS" OR "MEWT" OR "MEWTs" OR "MEOWS" OR "MEWC" OR "MERC")

2. TS=(early NEAR/2 (warning OR recognition OR surveillance) NEAR/1 (trigger* OR criteria OR score* OR system*))

3. TS=(develop* OR design* OR creat* OR build* OR contruct* OR validat* or evaluat*)

4. TS=(prognos* NEAR/1 (modelling OR modeling OR model OR models OR predict* OR performance OR tools OR ability OR accuracy OR probability OR risk OR factor*))

5. TS=("risk model*" OR "predict* the prognosis of" OR "predict* the risk of" OR "predict* the probability of" OR "candidate predictor*" OR "predictive clinical parameter*" or "descriptive model*")

6. TS=(probability NEAR/1 (modelling OR modeling OR model OR models))

7. TS=(predict* NEAR/1 (modelling OR modeling OR model OR models OR tools OR performance OR ability OR accuracy OR probability OR risk))

8. TI=((predict* OR prognos*) NEAR/1 (index OR nomogram))

9. #4 OR #5 OR #6 OR #7 OR #8

10. TS=((maternal OR maternity OR obstetric* OR perinatal OR pregnancy OR "antepartum" OR "ante-partum" OR "ante partum" OR delivery OR labour OR labor) NEAR/2 (morbidity OR death OR mortality OR "vital sign*" OR decompensation OR deteriorat$ OR complication* OR infection* OR sepsis OR "ICU admission" OR "critical care" OR "intensive care" OR "high dependency unit*" OR "HDU" OR "HDUs" OR "critical level 2"))

11. TS=(("post partum" OR "postpartum" OR "post-partum" OR "post natal" OR "postnatal" OR "post-natal" OR "post delivery" OR "postdelivery" OR "post-delivery") NEAR/2 (haemorrhage OR hemorrhage OR bleed* OR "blood loss"))

12. TS=("pre-eclampsia" OR eclampsia OR preeclampsia OR HELLP OR "acute fatty liver of pregnancy" OR "rupture of the uterus")

13. TS=((uterus OR uterine) NEAR/1 (ruptur* or perforat*))

14. #10 OR #11 OR #12 OR #13

15. #2 OR #9

16. #3 AND #14 AND #15

17. #1 OR #16

18. PY=(2000 OR 2001 OR 2002 OR 2003 OR 2004 OR 2005 OR 2006 OR 2007 OR 2008 OR 2009 OR 2010 OR 2011 OR 2012 OR 2013 OR 2014 OR 2015 OR 2016 OR 2017 OR 2018 OR 2019 OR 2020 OR 2021 OR 2022 OR 2023)

19. #17 AND #18

RESULTS = 1908 references
